# Prevalence of resistance mutations associated with integrase inhibitors in therapy-naive HIV-positive patients in Baoding, Hebei province, China

**DOI:** 10.3389/fgene.2022.975397

**Published:** 2022-09-14

**Authors:** Weiguang Fan, Xiaodong Wang, Yuchen Zhang, Juan Meng, Miaomiao Su, Xuegang Yang, Haoxi Shi, Penghui Shi, Xinli Lu

**Affiliations:** ^1^ Clinical Laboratory, The People’s Hospital of Baoding, Baoding, Hebei, China; ^2^ Infection Division, The People’s Hospital of Baoding, Baoding, Hebei, China; ^3^ Department of AIDS Research, Hebei Provincial Center for Disease Control and Prevention, Shijiazhuang, Hebei, China

**Keywords:** HIV, drug resistance mutations, integrase strand transfer inhibitors, genotype, baoding city

## Abstract

Antiretroviral therapy (ART) regimens containing integrase strand transfer inhibitors (INSTIs) are the recommended treatment for human immunodeficiency virus type 1 (HIV-1)-infected patients in the most recent guidelines in China. In this study, we investigated INSTI resistance mutations in newly diagnosed therapy-naive HIV-positive patients in Baoding City, Hebei Province (China) to provide guidance for implementing routine INSTI-associated HIV-1 genotypic resistance testing. Plasma samples were collected from HIV-1-infected patients without treatment at Baoding People’s Hospital from January 2020 to December 2021. The part of HIV-1 *pol* gene encoding integrase was amplified, sequenced, and analyzed for INSTI resistance. Clinical data including demographic data, CD4^+^ T cell counts, HIV-RNA loads, and resistance mutations were collected. Treatment-naïve HIV-1 patients (*n* = 131) were enrolled. We identified ten genotypes, and the predominant genotype was CRF01_AE in 67 patients (51.15%), CRF07_ BC in 39 patients (29.77%), subtype B in 11 patients (8.40%), and other subtypes (CRF68_01B, 3.82%; CRF55_01B, 1.53%, CRF80_0107, 1.53%; URFs 1.53%; and CRF103_01B, CRF59_01B, and CRF65_cpx, 1.4% each). Four major (E138A, R263k, G140S, and S147G) and three accessory (H51Y, Q146QL, and S153F) INSTI-resistance mutations were observed (genotype CRF01_AE, three patients; genotype B, one patient; and genotype CRF07_BC, one patient), resulting in different degrees of resistance to the following five INSTIs: raltegravir, elvitegravir, dolutegravir, bictegravir, and cabotegravir. The overall resistance rate was 3.82% (5/131). All INSTI-resistant strains were cross-resistant. The primary INSTI drug resistance rate among newly diagnosed HIV-infected patients in Baoding was low, but monitoring and research on HIV INSTI resistance should be strengthened in Baoding because INSTI-based regimen prescriptions are anticipated to increase in the near future.

## Introduction

Human immunodeficiency virus (HIV), the causative agent of acquired immune deficiency syndrome (AIDS), was first reported in 1981, and it quickly became a major epidemic threatening human health (Centers for Disease Control and Prevention, 1981). Today, antiretroviral therapy (ART) has transformed AIDS from a fatal disease into a treatable but currently incurable chronic disease, and there have been significant changes in the life expectancy of people living with HIV (Trickey et al., 2017). However, with the widespread application of antiretroviral drugs in clinical practice, HIV drug resistance has become an important factor affecting ART efficacy (Godfrey et al., 2017; Zhao et al., 2017). The HIV resistance rate to non-nucleoside reverse transcriptase inhibitors has increased significantly, and some areas have even experienced a high rate of transmissible resistance, leading to incomplete viral suppression and treatment failure (Wittkop et al., 2011; Clutter et al., 2016; World Health Organization, 2019). One of the newest antiretroviral drug classes is integrase strand transfer inhibitors (INSTIs), which target the HIV integrase enzyme and block incorporation of reverse transcribed proviral DNA into the host genome. Because they have excellent tolerability, minimal toxicity, high efficacy, and are easy to use, integrase inhibitors became preferred agents for treatment-naive or experienced patients, and they are a novel treatment option for acquired and transmitted resistance in combination with other HIV drug classes (Winans and Goff, 2020; Boyd and Donovan, 2013; NAMSAL ANRS 12313 Study Gro up Kouanfack et al., 2019; Durham and Chahine, 2021). The United States Food and Drug Administration (United States FDA) has approved the following five drugs for clinical use: dolutegravir (DTG), raltegravir (RAL), elvitegravir (EVG), bictegravir (BIC), and cabotegravir (CAB). Despite integrase inhibitors plays an effective role in antiretroviral action with a novel mechanism of action, resistance is inevitable (Jiang et al., 2016), and previously published studies have shown that there were important INSTIs resistance mutations in newly diagnosed HIV-1 patients in Spain, Canada, and the United States (Stekler et al., 2015; Ji et al., 2018; Casadellà et al., 2020; López et al., 2021). Major INSTIs resistance mutations have also been discovered in Yunnan (Deng et al., 2019) and Jiangsu (Yin et al., 2021) in China, and INSTIs resistance mutations were found in our neighboring provinces such as Henan (Yang et al., 2021), Beijing (Yu et al., 2022). Therefore, early monitoring of INSTIs drug resistance is of great significance for clinical development of HIV medication guidance and timely adjustment of medication regimens. However, there have been no previous reports on the spread of INSTIs drug resistance strains in Hebei. Therefore, this study was a preliminary analysis of primary integrase gene mutation and drug resistance in Baoding to provide a reference for preventing and treating HIV-1 patients in China.

## Materials and methods

### Study participants

Baoding People's Hospital is the designated hospital of ART and takes charge of anti-HIV therapy of all HIV-1-infected individuals in Baoding city. In this study, a total of 131 HIV-1-infected individuals were recruited in Baoding City before starting ART from January 2020 to December 2021. Their blood samples were collected in Baoding People's Hospital, and written informed consent was obtained from all subjects before blood collection. The baseline demographic characteristics were investigated using face-to-face interviews when we collected subjects’ blood samples. CD4^+^ T cell counts were determined using the BD FACSCount system (Becton Dickenson, CA, United States). Plasma HIV RNA levels were quantitatively tested using the Ampliform HIV-1 Monitor Test, version 1.5 (Roche, Cobas AmpliPrep/TaqMan 48, Switzerland). The detection limit threshold was <20 copies/ml.

### Nucleotide acid purification and polymerase chain reaction amplification

Viral RNA was purified using the QIAamp Viral RNA Mini Accessory Set (Qiagen, Duesseldorf, Germany), in accordance with the manufacturer’s instructions. The part of HIV-1 *pol* gene encoding integrase (HXB2: 4053-5214) was amplified in two steps using self-designed primers. For the first-round of reverse transcriptase-polymerase chain reaction (RT-PCR) amplification, we used the M-MLV 4 One-Step RT-PCR kit (Beijing Bomaide Gene Technology, Beijing, China), in accordance with the manufacturer’s instructions and using the following primers: INF12-2:5′-GCATTAGGRATYATTCARGCAC-3′ (outer l forward), INF12-1:5′-GGRATYATTCARGCACAACCAG-3′ (outer forward), and INR15-1: 5′-TGGGATRTGTACTTCYGARCTTA-3′ (outer reversal). Thermal cycling conditions for the first round of RT-PCR consisted of reverse transcription at 50°C for 45 min, inactivation at 95°C for 2 min, which was followed by three cycles of amplification at 95°C for 20 s, 50°C for 30 s, and 72°C for 2 min and 30 s. This was followed by 35 amplification cycles 95°C for 15 s, 50°C for 20 s, and 72°C for 2 min, with a final extension at 72°C for 10 min. Using the first-round RT-PCR product as a template, the second-round of PCR was performed using TaKaRa Premix Taq (TaKaRa Biotechnology, Dalian, China). Amplification was performed using the following primers: INF09:5′-TCTAYCTGKCATGGGTRCCAGCAC-3′ (inner forward) and INR18:5′-CATCCTGTCTACYTGCCACAC-3′ (inner reversal). The PCR conditions were as follows: a denaturing step at 95°C for 4 min, followed by three cycles at 95°C for 30 s, 55°C for 30 s, and 72°C for 2 min and 30s; then 35 amplification cycles at 95°C for 20 s, 55°C for 20 s, and at 72°C for 2 min; and a final extension at 72°C for 10 min. Positive products were detected using 1.2% agarose gel electrophoresis, and the positive products with the same size, as shown by the electrophoresis bands, were purified and sequenced using the Sanger sequencing technology by the Beijing Bomaide Gene Technology Co., Ltd. (Beijing, China). The sequencing primers were selected from the second round of amplification primers INF09, INR18 and KVL082: 5′-GGVATTCCCTATCAATCCCCAAAG-3′, KVL083: 5′-GAA​TAC​TGC​CAT​TTG​TAC​TGC​TG-3′.

### Sequence and drug-resistant mutation analysis

Raw sequences were assembled using Contig Express 9.1. Multiple sequence alignment was completed using ClustalW, and manual editing was performed using Bio-Edit 7.0 software. A neighbor-joining phylogenetic tree was constructed using the Kimura two-parameter model with 1000 bootstrap replicates using Molecular Evolutionary Genetic Analysis version 6.0 software (MEGA 6.0). HIV-1 subtypes were preliminarily analyzed using the online REGA HIV-1 Subtyping Tool 3.0 (http://dbpartners.stanford.edu:8080/RegaSubtyping/stanford-hiv/typingtool/) and identified using a neighbor-joining tree based on the HIV-1 integrase gene sequences. The Stanford HIV-1 drug resistance database (HIVdb version 9.0) was used to analyze the mutations. The Stanford HIVdb algorithm classifies the likelihood of INSTI resistance mutations being associated with resistance into “susceptible”, “potential low-level”, “low-level”, “intermediate”, and “high-level.” In our study, sequences containing low-level to high-level gene mutations were defined as resistance sequences. The related sequences were submitted to GenBank, and the accession numbers are ON529336 - ON529466.

### Statistical analysis

Statistical analysis was conducted using SPSS 21.0 (IBM Corp., Armonk, NY, United States). Means or frequencies were used to summarize demographic data. The differences of polymorphisms mutations according to subtypes were analyzed using chi-square test. All tests were two-tailed, and *p*-values <0.05 were considered statistically significant.

## Results

### Patient characteristics

**FIGURE 1:**
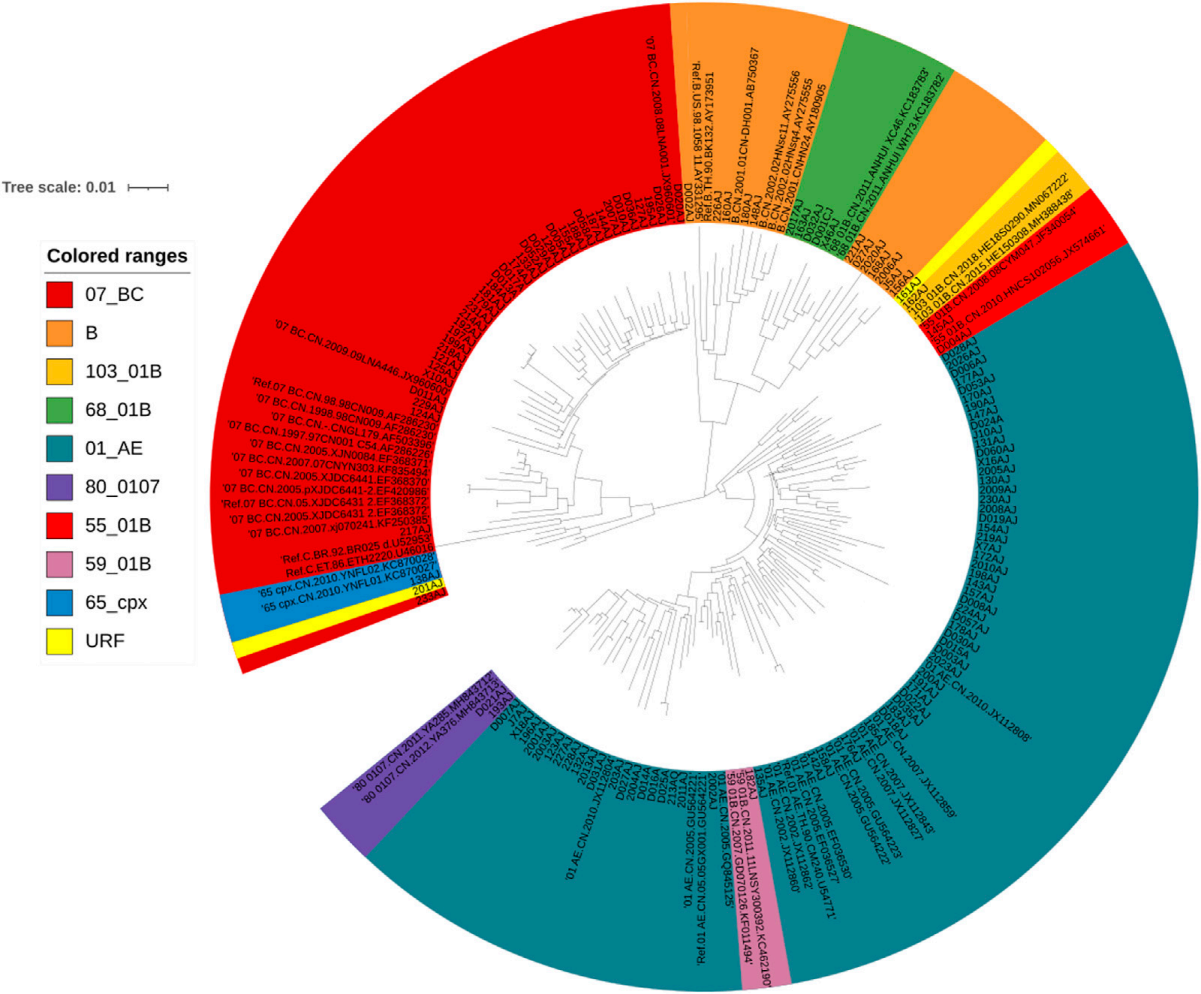
Phylogenetic tree analysis based on the HIV-1 integrase gene sequences. A neighbor-joining tree was constructed using MEGA 6.0 with 1000 bootstrap replicates, and were adjusted using the online itol (https://itol.embl.de/). The standard reference sequences of HIV-1 subtypes were downloaded from the HIV database (http://www.hiv.lanl.gov/content/index). Different subtypes are shown in using different colors listed in this figure.

One hundred thirty-one patients were evaluated in this study. Most were men (115/131, 87.79%), and the median age was 34 years (range, 18–76 years). The most common transmission routes were among men who have sex with men (MSM) (87.79%, 115/131) followed by heterosexual (HET) transmission (12.21%, 16/131). The median CD4^+^ T cell count was 166 cells/μl (range, 4–926 cells/μl). HIV-1 RNA loads ranged from 4.72 log10 RNA copies to 7.57 log10 RNA copies/ml (Table 1). Phylogenetic analysis of the integrase (IN) gene revealed that the sequenced strains could be divided into ten genotypes (Figure 1). The prevalence of each subtype was as follows: CRF01_AE (51.15%, 67/131), CRF07_BC (29.77%, 39/131), B (8.40%,11/131), and other subtypes (10.69%, 14/131).

**TABLE 1:** Clinical characteristics of patients at enrolment.

Variables	Patients N (%)
Age (years)	
18–50	113 (86.26)
51–76	18 (13.74)
Sex	
Male	115 (87.79)
Female	16 (12.21)
Transmission route	
MSM	115 (87.79)
Heterosexual	16 (12.21)
CD^4^T-cell count (cells/μL)	
<200	45 (34.35)
≥200	86 (65.65)
HIV-1 viral load (log_10_ RNA copies/mL)	
<5	66 (50.38)
≥5	65 (49.62)
Genotype	
CRF01_AE	67 (51.15)
CRF07_BC	39 (29.77)
B	11 (8.40)
CRF68_01B	5 (3.82)
CRF55_01B	2 (1.53)
CRF80_0107	2 (1.53)
URFs	2 (1.53)
CRF103_01B	1 (0.76)
CRF65_cpx	1 (0.76)
CRF59_01B	1 (0.76)

MSM, men who have sex with men; HIV, human immunodeficiency virus.

### INSTI resistance mutations

Drug resistance analyses showed that four major INSTI-resistance mutations (E138A, G140S, S147G, and R263K) and three accessory INSTI-resistance mutations (H51Y, Q146QL, and S153F) were detected in five patients. Information about INSTI resistance mutations corresponding to the specific drugs are listed in Table 2. To date, the CAB-associated resistance mutations have been listed in the HIVdb version 9.0. On the basis of this classification system, 96.18% of participants had viruses that were fully susceptible to five INSTIs. Low-level resistance to second-generation INSTIs DTG, BIC, and CAB were identified in 1.53% (2/131), 1.53% (2/131), and 2.29% (3/131) of participants, respectively. Intermediate-level resistance to all three second-generation INSTIs was 0.76% (1/131), and there was no high-level resistance. However, for both the first-generation INSTIs EVG, and RAL, low-grade resistance was 2.29% (3/131) and intermediate-level resistance was 0.76% (1/131). There was one case of high resistance to EVG. The overall INSTIs drug resistance rate was 3.82% (5/131) in this study.

**TABLE 2:** Characteristics of five ART-naive individuals with INSTI drug resistance mutations.

Patients CodeID	Age (years)	Sex	Transmission Route	Genotype	CD4 count (cells/?l)	Viral Load (log10 RNA copies/ml)	Integrase Mutations		Drug resistance				
							Major	accessory	BIC	DTG	CAB	EVG	RAL
130AJ	17	male	MSM	01_AE	416	5.34		H51Y	P	P	L	L	L
148AJ	56	male	MSM	B	542	4.71	E138A		P	P	P	L	L
178AJ	50	male	MSM	01_AE	362	5.58		S153F	L	L	L	L	P
192AJ	19	male	MSM	07_BC	868	4.37	R263K		I	I	I	I	L
2004AJ	26	male	MSM	01_AE	8	4.75	G140S,S147G	Q146QL	L	L	L	H	I

ART, anti-retroviral therapy; INSTI, integrase strand-transfer inhibitor; MSM, men who have sex with men; BIC, bictegravir; DTG, dolutegravir; CAB, cabotegravir; EVG, elvitegravir; RAL, raltegravir; P, potential low-level resistance; L, low-level resistance; I, intermediate resistance; H, high-level resistance.

### Natural polymorphisms of CRF01_AE and CRF07_BC strains at integrase resistance-related sites

Compared with the international standard strain HXB2 of subtype B, the CRF01_AE and CRF07_BC strains had more naturally occurring polymorphic variants in the encoded amino acids in the integrase gene coding region. The CRF01_AE strain had more than 50% mutation rates at K14R, V31I, I72V, T112V, T124A, T125A, G134N, I135V, K136Q, D167E, V201I, L234I/S, and S283G, while the CRF07_BC strain had K42Q, L101I, T112V, T124A, T125A, I135V, K136Q, V201I, R269K, D278A, and S283G have a mutation rate of more than 50%. There were statistically significant differences between the two genotype mutation rates at 14 polymorphic variants K14R, K42Q, I72V, L101I, T125A, G134N, I135V, K136Q, D167E, V201I, L234I, L234S, R269K, and D278A (*P* < 0.05; Table 3).

**TABLE 3:** Natural polymorphisms of CRF01_AE and CRF07_BC strains at integrase resistance-related sites.

polymorphic variants	CRF01_AE		CRF07_BC		P	polymorphic variants	CRF01_AE		CRF07_BC		*P*
	N	%	N	%			N	%	N	%	
K14R	45	67.16	10	25.64	<0.001	I135V	60	89.55	28	71.79	0.019
V31I	42	62.69	18	46.15	0.098	K136Q	62	92.54	30	76.92	0.022
K42Q	5	7.46	28	71.79	<0.001	D167E	42	62.69	5	12.82	<0.001
I72V	34	50.75	6	15.38	<0.001	V201I	67	100	33	84.62	0.001
L101I	18	26.87	30	76.92	<0.001	L234I	62	92.54	7	17.95	<0.001
T112V	63	94.03	34	87.18	0.222	L234S	2	2.99	24	61.54	<0.001
T124A	64	95.52	34	87.18	0.117	R269K	5	7.46	24	61.54	<0.001
T125A	65	97.01	31	79.49	0.003	D278A	6	8.96	27	69.23	<0.001
G134N	61	91.04	8	20.51	<0.001	S283G	61	91.04	32	82.05	0.173

Note: The differences of polymorphisms mutations according to subtypes were analyzed using chi-square test.

## Discussion

Since RAL (the first INSTI) was approved by the US FDA in 2007, HIV treatment has entered a new era. ART regimens containing INSTIs have become the recommended treatment for HIV-1-infected patients in the Chinese Guidelines for Diagnosis and Treatment of HIV/AIDS (2021) in China (AIDS and Hepatitis C Professional Group, 2021). However, with the widespread use of INSTIs, INSTI-related drug resistance mutations have gradually emerged. This study monitored the prevalence of resistance mutations associated with HIV INSTI in ART-naïve patients in Baoding from January 2020 to December 2021. Four major INSTI-resistant mutations were detected in five patients, including E138A, G140S, S147G, and R263K as well as three accessory INSTI-resistant mutations H51Y, Q146QL, and S153F, which cause varying degrees of resistance in the following five INSTIs: BIC, DTG, CAB, EVG, and RAL (Table 2). The prevalence of INSTI resistance was 3.82% (5/131). This INSTI resistance prevalence was higher than that in Jiangsu (0.76%) (Yin et al., 2021), Henan (1.7%) (Yang et al., 2021), and Beijing (0.34%) in China (Yu et al., 2022) as well as in Italy (0.2%) (Rossetti et al., 2018), Austria (2.3%) (Zoufaly et al., 2017), and Uganda (1.2%) (McCluskey et al., 2021), but lower than that in Yunnan (5.7%) (Deng et al., 2019) and Poland (8.3%) (Parczewski et al., 2012) and close to that in Korea (3.4%) (Jeong et al., 2019), which suggests that it is necessary for us to strength the surveillance of INSTIs resistance in order to control and prevent the spread of HIV-1 resistant strains.

In this study, all INSTI-resistant strains were resistant to EVG, and one patient was highly resistant to EVG. In the subtype distribution, there were three patients with the CRF01_AE genotype, which is carried in the H51Y, S153SF, G140S, S147SG, and Q146QL mutations, and it had high, intermediate, and low levels of resistance to five INSTIs. One patient who had subtype B carried E138A and had low resistance to RAL and EVG, and one patient with the CRF07_BC subtype carried the R263K mutation and had intermediate resistance to BIC, DTG, CAB, and EVG but low resistance to RAL. R263K is selected *in vitro* using EVG, DTG, and BIC and in patients receiving DTG. It reduces DTG and BIC susceptibility approximately two-fold and EVG susceptibility to a greater extent (https://hivdb.stanford.edu/dr-summary/comments/INSTI.[OL]/). E138A is non-polymorphic mutation that is selected-for in patients receiving RAL, EVG, and DTG. Alone, this non-polymorphic mutation does not reduce INSTI susceptibility, but when it occurs in combination with the Q148 mutations, it is associated with high-level resistance to RAL and EVG and an intermediate reduction to DTG and BIC susceptibility (https://hivdb.stanford.edu/dr-summary/comments/INSTI.[OL]/). However, this study showed that none of these mutations occurred in the E138A and Q148 mutation combinations. In the past years, INSTIs have never been included in the free ART regimens in Hebei. We infer that HIV-1 INSTI resistant strans should be introduced into Hebei from other areas.

In this study, many amino acid polymorphisms that are different from the standard B subtype strains were detected in both the CRF01_AE and CRF07_BC strains, which have also been reported in untreated persons who are infected with other genotype strains in other countries (Brado et al., 2018; Alaoui et al., 2019; Huang et al., 2019; Lai et al., 2021). Although these polymorphic variants were encountered in more than half of the studied participants, other polymorphic mutations that probably have no effect on INSTIs susceptibility were reported in previous *in vitro* studies, and clinical trials showed different frequencies (Kobayashi et al., 2011; Casadellà et al., 2015). However, naturally occurring polymorphisms impact the intasome complex stability and may, therefore, contribute to the overall potency against INSTIs and natural polymorphisms, while subtype-specific differences may influence the effect of individual treatment regimens (Brado et al., 2018). The differences of polymorphic variants between CRF01_AE and CRF07_BC suggest that the disease progress of CRF01_AE is faster than CRF07_BC (Ye et al., 2022).

There are some limitations to our study, such as using only the IN gene in the genotyping accuracy and CRF identification. The lacking of reverse transcriptase and protease resistance data not only affects genotyping accuracy but also the assessment of resistance to first-line regimens. Additionally, the relatively small number of samples obtained for our analysis limits our ability to definitively provide frequency analysis for INSTI resistance mutations. Study results are also limited because Sanger sequencing was used instead of next-generation sequencing, which allows minority variants to be reported. In our future study, we will increase the sample size and sequence the whole length *pol* gene using more sensitive techniques such as next-generation sequencing methods to minimize the limitations.

## Conclusion

The respective prevalence rate of INSTI major resistance mutations in ART-naive patients in Baoding was 3.82%. We think that the surveillance of INSTI resistance should be recommended before regarding treatment regimens containing INSTI are planned in Baoding city. The study is small and a more clear picture of INSTI resistance in China should be evaluated in a larger national study.

## Data Availability

The datasets presented in this study can be found in online repositories. The names of the repository/repositories and accession number(s) can be found in the article/supplementary material.

## References

[B1] AIDS and Hepatitis C Professional Group. (2021) The Chinese guidelines for diagnosis and treatment of HIV/AIDS (2021)[J]. Chin. J. AIDS STD 27(11), 1182–1201.

[B2] Alaoui N. El Alaoui M. A. El Annaz H. Farissi F. Z. Alaoui A. S. El Fahime E. (2019). HIV-1 integrase resistance among highly antiretroviral experienced patients from Morocco. Intervirology 62 (2), 65–71. 10.1159/000501016 31307042

[B3] Boyd M. A. Donovan Donovan B. (2013). Antiretroviral therapy: Dolutegravir sets SAIL(ING). Lancet (London, Engl. 382, 664–666. 10.1016/S0140-6736(13)61456-7 23830358

[B4] Brado D. Obasa A. E. Ikomey G. M. Cloete R. Singh K. Engelbrecht S. (2018). Analyses of HIV-1 integrase sequences prior to South African national HIV-treatment program and available of integrase inhibitors in Cape Town, South Africa.Erratum in. Sci. Rep.Sci Rep. 88 (11), 47096262. 10.1038/s41598-018-22914-5 PMC5856838 29549274

[B5] Casadellà M. Santos J. R. Noguera-Julian M. Mican-RiveRa R. Domingo P. AntelA A. (2020). Primary resistance to integrase strand transfer inhibitors in Spain using ultrasensitive HIV-1 genotyping. J. Antimicrob. Chemother. 75 (12), 3517–3524. 10.1093/jac/dkaa349 32929472

[B6] Casadellà M. van Ham P. M. Noguera-Julian M. vAn Kessel A. Pou C. Hofstra L. M. (2015). Primary resistance to integrase strand-transfer inhibitors in Europe. J. Antimicrob. Chemother. 70 (10), 2885–2888. 10.1093/jac/dkv202 26188038

[B7] Centers for Disease Control and Prevention (1981). Pneumocystic pneumonia-los angeles [J]. MMWR Morb. Mortal. Wkly. Rep. (3021), 1–3. 6798402

[B8] Clutter D. S. Jordan M. R. Bertagnolio S. Shafer R. W. (2016). HIV-1 drug resistance and resistance testing. Infect. Genet. Evol. 46, 292–307. 27587334 10.1016/j.meegid.2016.08.031 PMC5136505

[B9] Deng X. M. Liu J. F. Zhang M. (2019). Mutations of primary integrase gene resistance of HIV/AIDS patients in Yunnan province. Clin. J. AIDS STD 25 (4), 327–341.

[B10] Durham S. H. Chahine E. B. (2021). Cabotegravir-Rilpivirine: The first complete long-acting injectable regimen for the treatment of HIV-1 infection. Ann. Pharmacother. 55 (11), 1397–1409. 10.1177/1060028021995586 33593093

[B11] Godfrey C. Bobkova M. Boucher C. Ravasi G. Chen P. Zhang F. (2017). Regional challenges in the prevention of human immunodeficiency virus drug resistance. J. Infect. Dis. 216 (9), S816–S819. 10.1093/infdis/jix408 28968824 PMC5853282

[B12] Huang X. T. Sun Z. S. An M. H. Zhao L. Wang L. Ding H. B. (2019). Primary drug resistance to integrase inhibitors among newly diagnosed HIV infected patients in Shenyang city. Chin. J. Clin. Lab. Sci. 10, 721–725.

[B13] Jeong W. Jung I. Y. Choi H. Kim J. H. Seong H. Ahn J. Y. (2019). Integrase strand transfer inhibitor resistance mutations in antiretroviral therapy-naive and treatment-experienced HIV patients in South Korea. AIDS Res. Hum. Retroviruses 35, 213–216. 10.1089/AID.2018.0213 30229661

[B14] Ji H. Patterson A. Taylor T. Rank C. Halverson J. Capina R. (2018). Prevalence of primary drug resistance against HIV-1 integrase inhibitors in Canada. J. Acquir. Immune Defic. Syndr. 78 (1), e1–e3. 10.1097/QAI.0000000000001649 29424788 PMC5902129

[B15] Jiang J. Xu X. Guo W. Su J. Huang J. Liang B. (2016). Dolutegravir(DTG, S/GSK1349572) combined with other ARTs is superior to RAL- or EFV-based regimens for treatment of HIV-1 infection: A meta-analysis of randomized controlled trials. AIDS Res. Ther. 13 (1), 30. 10.1186/s12981-016-0115-x 27617024 PMC5016965

[B16] Kobayashi M. Yoshinaga T. Seki T. Wakasa-Morimoto C. Brown K. W. Ferris R. (2011). *In vitro* antiretroviral properties of S/GSK1349572, a next-generation HIV integrase inhibitor. Antimicrob. Agents Chemother. 55 (2), 813–821. 10.1128/AAC.01209-10 21115794 PMC3028777

[B17] NAMSAL ANRS 12313 Study Group, Kouanfack C. Mpoudi-Etame M. Omgba Bassega P. Eymard-Duvernay S. Leroy S. (2019). Dolutegravir-based or low-dose efavirenz-based regimen for the treatment of HIV-1. N. Engl. J. Med. Overseas. Ed. 381, 816–826. 10.1056/nejmoa1904340 31339676

[B18] Lai J. Liu Y. Han X. Huang A. Lin J. Ao W. (2021). Low frequency of integrase inhibitor resistance mutations among therapy-naïve HIV patients in southeast China. Drug Des. devel. Ther. 15, 889–894. 10.2147/DDDT.S286863 PMC7924127 33679129

[B19] López P. Tirado G. Arias A. Sanchez R. Rodriguez-Lopez E. R. Rivera-Amill V. (2021). Short communication: Integrase strand transfer inhibitors drug resistance mutations in Puerto Rico HIV-positive individuals. Int. J. Environ. Res. Public Health 18 (5), 2719. 10.3390/ijerph18052719 33800269 PMC7967446

[B20] McCluskey S. M. Kamelian K. Musinguzi N. Kigozi S. Boum Y. Bwana M. B. (2021). Pre-treatment integrase inhibitor resistance is uncommon in antiretroviral therapy-naive individuals with HIV-1 subtype A1 and D infections in Uganda. AIDS 35 (7), 1083–1089. 10.1097/qad.0000000000002854 33635845 PMC8102316

[B21] Parczewski M. Bander D. Urbańska A. Boron-Kaczmarska A. (2012). HIV-1 integrase resistance among antiretroviral treatment naive and experienced patients from Northwestern Poland. BMC Infect. Dis. 12, 368. 10.1186/1471-2334-12-368 23259737 PMC3547692

[B22] Rossetti B. Di Giambenedetto S. Torti C. Postorino M. C. Punzi G. SaladiniF. (2018). Evolution of transmitted HIV-1 drug resistance and viral subtypes circulation in Italy from 2006 to 2016. HIV Med. 19, 619–628. 10.1111/hiv.12640 29932313

[B23] Stekler J. D. McKernan J. Milne R. Tapia K. A. Mykhalchenko K. Holte S. (2015). Lack of resistance to integrase inhibitors among antiretroviral-naive subjects with primary HIV-1 infection, 2007-2013. Antivir. Ther. 20 (1), 77–80. 10.3851/IMP2780 24831260 PMC4312242

[B24] Trickey A. May M. T. Vehreschild J-J Obel N. Gill M. J. Crane H. M. (2017). Survival of HIV-positive patients starting antiretroviral therapy between 1996 and 2013: A collaborative analysis of cohort studies. Lancet. HIV 4, e349–e356. 10.1016/S2352-3018(17)30066-8 28501495 PMC5555438

[B25] Winans S. Goff S. P. (2020). Mutations altering acetylated residues in the CTD of HIV-1 integrase cause defects in proviral transcription at early times after integration of viral DNA. PLoS Pathog. 16, e1009147. 10.1371/journal.ppat.1009147 33351861 PMC7787678

[B26] Wittkop L. Günthard H. F. de Wolf F. Dunn D. Cozzi-Lepri A. de Luca A. (2011). Effect of transmitted drug resistance on virological and immunological response to initial combination antiretroviral therapy for HIV (EuroCoord-CHAIN joint project): A European multicohort study. Lancet. Infect. Dis. 11 (5), 363–371. 10.1016/S1473-3099(11)70032-9 21354861

[B27] World Health Organization (2019). HIV drug resistance report 2019. Geneva: World Health Organization.

[B28] Yang Z. Yang X. Deng X. Wei S. Liu J. Ma J. (2021). Prevalence of integrase strand transfer inhibitor (INSTIs) resistance mutations in Henan Province, China (2018-2020). Infection 49 (6), 1195–1202. 10.1007/s15010-021-01668-9 34279816

[B29] Ye J. Chen J. Wang J. Wang Y. Xing H. Yu F. (2022). CRF07_BC is associated with slow HIV disease progression in Chinese patients. Sci. Rep. 12 (1), 3773. 10.1038/s41598-022-07518-4 35260599 PMC8904811

[B30] Yin Y. Q. Lu J. Zhou Y. Shi L. E. Yuan D. F. Chen J. S. (2021). Drug resistance to HIV-1 integrase inhibitors among treatment-naive patients in Jiangsu, China. Biomed. Environ. Sci. 34 (5), 400–403. 10.3967/bes2021.053 34059178

[B31] Yu F. Li Q. Wang L. Zhao H. Wu H. Yang S. (2022). Drug resistance to HIV-1 integrase inhibitors among treatment-naive patients in beijing, China. Pharmgenomics. Pers. Med. 15, 195–203. 10.2147/PGPM.S345797 35300056 PMC8922317

[B32] Zhao Y. Zhang M. Shi C. X. Huang J. Zhang F. Rou K. (2017). Mortality and virological failure among HIV-infected people who inject drugs on antiretroviral treatment in China: An observational cohort study. Drug Alcohol Depend. 170, 189–197. 10.1016/j.drugalcdep.2016.11.011 27987476 PMC6301141

[B33] Zoufaly A. Kraft C. Schmidbauer C. Puchhammer-Stoeckl E. (2017). Prevalence of integrase inhibitor resistance mutations in Austrian patients recently diagnosed with HIV from 2008 to 2013. Infection 45, 165–170. 10.1007/s15010-016-0936-5 27530391

